# Factors associated with exclusive breastfeeding for the first six months among caregivers of children under five years in northern Ghana: A cross-sectional study

**DOI:** 10.1371/journal.pgph.0003887

**Published:** 2024-11-14

**Authors:** Gretchen H. Thompson, Eunice Sefa, Ashwini S. Deshpande, Ransford Mensah, Rachel Lenzi-Weisbecker, Rose Wilder, Thad Pennas, Andres Martinez, Kingsley K. Boadi, Adnan Abdul-Hamid, Godwin Asabire, Dacosta Aboagye, Eliasu Yakubu, Abdulai Abubakari, David Yao Mensah

**Affiliations:** 1 Division of Global Research, Family Health International 360, Durham, North Carolina, United States of America; 2 Ghana Accelerating Social and Behavior Change Project, Family Health International 360, Accra, Ghana; 3 Division of Human Development, Family Health International 360, Durham, North Carolina, United States of America; 4 Ghana Ministry of Health, Health Promotion Division, Accra, Ghana; 5 Saha Consulting and Services Limited, Accra, Ghana; 6 School of Public Health, Department of Global and International Health, University for Development Studies, Accra, Ghana; 7 Tally Graduate School of Dominion University College, Accra, Ghana; Hiroshima University, JAPAN

## Abstract

Despite consistent evidence highlighting the benefits of exclusive breastfeeding (EBF) for the first six months, EBF duration sometimes falls short of six months or exceeds it. This research seeks to explore factors influencing the practice of optimal duration of EBF and identify factors associated with suboptimal EBF durations. A cross-sectional survey was conducted in 16 districts across four Northern regions of Ghana with 2000 caregivers of children under five years old. The analysis specifically focused on a subset of 1761 biological mothers. Logistic regression was conducted to discern socio-demographic, care-seeking, and behavioral determinants influencing the practice of EBF for a six-month duration. Additionally, multinomial logistic regression was used to identify factors associated with suboptimal EBF durations, categorized as less than and beyond six months. All biological mothers breastfed their youngest child, varying in durations: 75% exclusively breastfed for six months, 19.2% for less than six months, and 5.9% for beyond six months. Several behavioral determinants influenced EBF for six months, with biological mothers ’ confidence in their ability to EBF (aOR: 6.8 95% CI, 4.13–11.33), willingness to recommend EBF practice to friends and family (aOR: 10.5 95% CI, 4.13–26.62) and perception of practicing EBF as normative in the community showing statistically significant associations (aOR: 6.3, 95% CI, 4.41–9.10). Education and religion of biological mothers were also significantly associated with EBF for six months. While there was overlap in behavioral factors associated with EBF for less than and beyond six months, the socio-demographic characteristics of biological mothers associated with these suboptimal durations of EBF differed. Among the factors included in this study, social norms, caregiver confidence, and approval of EBF were key factors influencing the recommended duration of EBF practices. These findings highlight the importance of community and cultural contexts in shaping biological mothers’ behavior and emphasize the need to address socio-cultural barriers and facilitators that influence EBF practices, as well as empower biological mothers to adopt and sustain these practices.

## Introduction

Exclusive breastfeeding (EBF) is the practice of feeding an infant with breast milk only, without any additional liquids or solid foods. EBF for the first six months, without caregivers offering pre-lacteal feedings, is an important accelerator behavior because of its potential to directly impact the leading causes of child mortality [1, 2]. First and foremost, it provides infants with the ideal nutrition during the first six months of life, offering essential nutrients, antibodies, and immune-boosting factors that help protect them from common illnesses and infections. EBF has been shown to greatly enhance child health and nutrition outcomes in low and middle-income countries. The World Bank estimates that if breastfeeding were scaled up to universal levels globally, over 800,000 infant lives would be saved [3]. Breast milk specifically meets the nutritional needs of babies, offering the ideal balance of proteins, fats, vitamins, and minerals that promote healthy growth and development. It not only protects against a myriad of common childhood illnesses, reducing infant mortality rates but also bolsters their immune system, safeguarding them against infections and malnutrition, which are prevalent in resource-constrained settings. Moreover, EBF fosters the bond between mothers and infants and supports cognitive development, setting the foundation for a lifetime of improved health and well-being, making it a vital component in addressing child health and nutrition challenges [3].

According to a nationally representative survey in Ghana, only 53.1% of children aged 0–5 months were fed exclusively with breastmilk during the previous day [4]. In the Northern region of Ghana, one study revealed that while nearly all mothers exclusively breastfed their child for six months, 42.1% exclusively breastfed until eight months, and 16.8% exclusively breastfed beyond eight months [5]. EBF for the first six months of a newborn’s life is one of the prioritized behaviors promoted through the Accelerating Social and Behavior Change (ASBC) Activity, a five-year project (2021–2026) funded by the United States Agency for International Development (USAID)/Ghana and implemented by a consortium of partners led by FHI 360. The ASBC Activity primarily focuses on the 17 zone of influence (ZOI) districts in Northern Ghana where severe health disparities persist compared to national trends. The integrated health project seeks to increase uptake of EBF through community-level social and behavior change (SBC) interventions, primarily using communication channels like interpersonal communication, mass media (e.g., community, regional and national radio stations, TV, social and digital media), and various innovative and traditional approaches.

Previous research suggests that a multitude of factors across individual levels, health systems, work environments, communities, households, and structural levels influence the practice of EBF [6]. On an individual level, biological maternal factors like age, obesity, cesarean section delivery, and birth complications, alongside psychological factors such as anxiety regarding the infant’s health and low self-efficacy, were linked to a higher likelihood of early discontinuation of breastfeeding [6, 7]. At the health systems level, access to maternity care facilities adhering to the ten steps to successful breastfeeding–such as educating expectant mothers on the advantages of breastfeeding, promoting early breastfeeding initiation, providing expert lactation support, refraining from offering pre-lacteal feeds or formula supplements during hospital stays, and facilitating rooming-in practices–have shown to positively influence the duration of EBF [8]. Lower household income and parental education were identified as factors that hindered EBF [9]. At the structural level, factors such as media campaigns endorsing commercial milk formula and the lack of national policies supporting family-friendly childcare initiatives hindered the establishment of a conducive environment for promoting EBF [10].

Individual, social, and structural factors previously found to influence EBF for the first six months in Ghana, include household wealth, maternal education, prior antenatal care (ANC) attendance, knowledge of EBF, and the place of delivery [11–13]. Healthcare workers in Ghana are generally supportive of EBF, and prior attendance at ANC is associated with higher rates of EBF. However, lower-quality ANC visits have been associated with poorer infant feeding practices, emphasizing the importance of improving the quality of healthcare services [12]. An evaluation of an urban hospital in Ghana, recognized as baby-friendly, revealed that the overall adherence to the ten steps to successful breastfeeding was moderate. However, compliance was low in several key steps, including staff training, early initiation of breastfeeding, and breastfeeding policies [14]. The research on breastfeeding has shown improvement in breastfeeding practices due to the mother’s high self-confidence in her ability to practice and master breastfeeding satisfactorily and has identified it as an essential factor in maternal ideation regarding EBF [15]. Another study in Ghana identified knowledge gaps and misconceptions surrounding EBF as important drivers that may prevent EBF for the first six months [13]. Grandmothers and traditional birth attendants, who hold influence over breastfeeding mothers, have also been found to demonstrate poor knowledge of EBF, and they sometimes provide conflicting advice or feed babies substances like water or animal milk [11]. This highlights the importance of community and family involvement in EBF interventions, as well as the need to dispel common myths and misconceptions around breastfeeding and child development and nutritional needs [16–18]. In 2000, the Government of Ghana enacted the National Breastfeeding Promotion Regulation (NBPR), categorizing breast milk substitutes as "designated products." While NBPR regulates various aspects concerning these products, it lacks specific provisions for marketing processed commercial complementary foods for children aged six months and older. This regulatory gap allows companies to exploit the situation, potentially misleading caregivers into initiating complementary feeding prematurely [19].

Mother’s daily household chores or employment-related needs served as barriers to continued breastfeeding [12, 20]. In particular, the demands of cooking daily meals and traveling long distances to fetch water were significant factors hindering a woman’s capacity to exclusively breastfeed [20]. Employed mothers indicate that they are unable to bring their baby to the workplace for breastfeeding due to their demanding schedules [12, 20]. Consequently, within the group of employed mothers, one study revealed a low prevalence of EBF (10.3%), despite their comprehensive understanding of its significance and previous early initiation of breastfeeding. Although most working mothers typically benefit from a three-month maternity leave, a staggering 69% lacked employer support for continued breastfeeding after this period [12]. Women with maternity leaves less than three-months were less likely to EBF.

While EBF for the first six months is important for a child’s health, the necessity for introducing complementary foods arises after the sixth-month period since breast milk alone becomes inadequate to meet the nutritional requirements of infants. Infants should be exposed to complementary foods at six months while continuing to breastfeed up to two years or beyond [21, 22]. Complementary foods consist of milk formula or animal milk, animal-derived products such as meat, fish, or eggs, along with fruits and vegetables, as well as pulses, nuts, and seeds. The predominant emphasis in existing research in Ghana has been on identifying factors influencing EBF for six months. Consequently, these studies do not include the characteristics of caregivers who exclusively breastfeed beyond six months without incorporating the essential and timely introduction of complementary feeding in a child’s diet [17]. While this paper seeks to examine the factors associated with the recommended optimal duration of EBF, i.e., for the first six months, it also importantly characterizes factors that shape women’s decision to follow suboptimal EBF practices, both below and beyond the six-month optimal threshold.

Based on our review of the literature and data collected as a part of ASBC baseline assessment, we examine the effects of social norms, self-efficacy, attitudes, facility birth attendance, ANC attendance, and equitable gender attitudes on adopting the practice of EBF for the first six months. We also explore the effect that religion may have on EBF, and we also examine the effects of sociodemographic variables including employment, education, and wealth. This paper analyzes data from the ASBC baseline assessment, implemented by FHI 360 in Northern Ghana. Overall, the ASBC Activity aims to promote and maintain the uptake of healthy behaviors and practices, emphasizing family planning/reproductive health (FP/RH), malaria prevention and treatment, maternal, newborn, and child health (MNCH), community and household-level water, sanitation, and hygiene (WASH) practices, nutrition, and addressing public health emergencies, such as COVID-19.

## Methods

This analysis utilizes baseline cross-section household survey data, which was collected in August-September 2022 by a research firm based in Northern Ghana—Saha Consulting and Services in the regions/districts where the ASBC Activity will be implemented over five years from 2021 to 2026. The study was reviewed and approved by the Ghana Health Service Ethics Review Committee (ERC). Data collectors’ training was conducted from August 29, 2022 to September 1, 2022 and the data was collected from September 2, 2022 to September 10, 2022.

### Study setting

The study site includes four regions in Northern Ghana, namely Northern, North East, Upper East, and Upper West. The Upper West region in Northern Ghana has the highest poverty rate (70.7%), followed by the Northern region (50.4%) and the Upper East region (44.4%), according to the poverty line set at 1,314 GHS per adult per year, as per the Ghana Living Standards Survey of 2013 [23]. Overall, Northern Ghana faces challenges such as high rates of under-five malnutrition, maternal mortality, and limited access to basic healthcare services [24]. In Northern Ghana, 33% of children in the Northern region are stunted compared to a national average of 19 percent. These challenges highlight the need for evidence-based social and behavior change interventions to improve health outcomes in Northern Ghana, including factors that influence EBF for the first six months.

### Sampling

Stratified random sampling techniques were employed to collect the baseline data. A list of enumeration areas (EAs) in each of the 16 ASBC implementation districts was obtained from the Ghana Statistical Service’s 2021 Population and Housing Census. Only EAs with a minimum of 100 and a maximum of 500 households were included in the sampling. The EAs were then stratified by rural and urban locations. In all, 64 EAs (32 each of rural and urban) were sampled for data collection. The study team added an additional EA in each of the 16 districts to compensate for a skip pattern error detected during data collection which reduced the number of responses for non EBF survey topics investigated. In total, 80 EAs were sampled. About 25 households were randomly selected from each of the selected EAs for the baseline survey. In the final stage of sampling, women (15–49 years) and men (>15 years) caregivers of children under five years of age were selected as the primary respondents by the interviewer. In cases where multiple eligible respondents were identified as biological mothers within the household, one respondent was chosen at random.

Written consent was obtained but this did not include any personal identifiers of respondents to protect participant privacy. For adolescents/young persons (less than 18 years), parental/guardian permission was requested, followed by child assent. For young persons under 18 years of age who were married, pregnant, or mothers, personal consent on account of being emancipated minors (children who have assumed adult responsibilities) was sought.

COVID-19 precautions were based on interim guidance for household-level data collection during the COVID-19 pandemic released by the World Health Organization in October 2020. Measures included providing survey team members with three surgical masks daily, along with hand sanitizer to minimize the risks of potential transmission and infection. Interviews were conducted outdoors in open, shaded areas with physical distancing, or indoors with proper distancing and ventilation. The congregation of others around interview sites was prevented.

### Data

The baseline dataset included primary caregivers (n = 2000) responsible for children under the age of five, including both women and men fulfilling the role of primary caregivers. However, the analysis specifically focused on the subset of 1761 biological mothers of children under five. The baseline dataset included information on EBF practice as reported by the mothers, along with data on socio-demographic characteristics, care-seeking behaviors, and behavioral determinants related to EBF (such as perceived social norms, self-efficacy, and approval of health practices). The questions regarding care-seeking and behavioral determinants were framed concerning the most recent pregnancy or childbirth.

### Study variables

The outcome variable in this study, EBF, was determined by the question: ’Following your most recent childbirth, how many months did you wait before introducing anything other than breastmilk, such as formula, plain water, cereals, or other mashed foods?’ Respondents provided their answers in terms of months. These responses were dichotomized: 1 indicating mothers who reported waiting for an exact duration of six months before introducing liquids and foods other than breastmilk (EBF for six months), and 0 for mothers who reported waiting for less than or more than 6 months (EBF less than or beyond six months). The outcome variable was also converted to a categorical variable, with 1 indicating mothers who reported waiting for six months before introducing liquids and foods other than breastmilk, 0 for mothers who reported waiting for less than six months, and 2 for mothers who reported waiting for more than six months.

The independent variables were grouped into socio-demographic factors, care-seeking behaviors, and behavioral determinants. Sociodemographic characteristics included the caregiver’s residential location (region and type of residence [rural/urban]), age, religion, educational background, employment status, household asset index, and gender equitable index. The household asset index, generated using principal component analysis, consisted of eight assets: drinking water source, refrigerator, car, motorbike, television, bank account, phone, and toilet. The gender equitable index was a composite of nine Likert-type gender-equitable statements, with scores ranging from 9 to 54. This variable, representing the total, was divided into four 25th percentiles, from the most desirable group (i.e., first quartile) to the least (i.e., 4th quartile). The gender statements were adapted from the Traditional Values sub-scale of the Healthy Relationship Assessment Tool [25].

The care-seeking behavior-related factors included the completion of at least four ANC visits at a healthcare facility during the last pregnancy. It also included a variable indicating whether the caregiver delivered at a healthcare facility during the last delivery.

Behavioral factors influencing EBF for the first six months included perceptions of when other caregivers in the community introduced foods other than breastmilk (perceived descriptive norm), mothers’ self-efficacy to exclusively breastfeed for the first six months, and positive attitudes toward (i.e., approval) EBF for the first six months based on whether the mothers would encourage friends or family members to wait six months before introducing any liquids or other foods, including water, formula, cereals, or mashed foods. We used the following questions to examine the effects of these behavioral factors:

- In your community, at what age do most families introduce babies to foods other than breastmilk?
- If you [your partner] were to have a baby in the future, how confident are you in your ability to exclusively breastfeed (give only breastmilk) your baby for six months if you [she] wanted to do so?
- If a friend or family member were to have a baby in the future, would you encourage her to wait six months before introducing any liquids or other foods—including water, formula, cereals, or mashed foods?

### Statistical analysis

The data analysis utilized Stata software (v18.0) for Windows. Initially, we examined associations between the dichotomized variable indicating EBF up to six months and independent variables through univariate logistic regressions. Significant variables from the univariate tests were incorporated into a multivariate logistic regression model. A series of nested models were developed to identify theoretical groupings of sociodemographic, care-seeking, and individual behavioral determinants associated with EBF behavior. We conducted a Wald test to determine whether a set of independent variables within each theoretical grouping collectively exhibited ’significance’ for a model. Furthermore, we identified the independent variable associated with EBF for the first six months. Throughout the analyses, a p-value less than 0.10 using a two-tailed t-test was considered statistically significant. We chose this threshold given that our hypothesized relationships between most independent and dependent variables are directional and thus suitable for a one-tailed t-test.

Additionally, we performed multinomial logistic regression to understand which factors were associated with suboptimal breastfeeding behaviors, i.e., EBF for less than or beyond six months. The reference category for the outcome in this multinomial logistic regression was EBF for six months. We investigated whether factors differed for those who exclusively breastfed for less than six months and those who continued EBF beyond six months. In the multivariate multinomial logistic regression, approval of EBF by biological mothers was excluded from the model due to a zero-cell size for those who breastfed beyond six months but did not approve of EBF. However, all other factors were included in the model.

The statistical analysis incorporated appropriate adjustments for the sampling weights and complex sampling design. The sampling weight was computed as 1/p, where p was the probability of selecting a household in an EA. The findings are representative of the EAs selected in the sample.

## Results

### Sample description

The sample comprised 1761 biological mothers. The summary of socio-demographic characteristics of the biological mothers and the outcome variable is presented in Tables 1 and 2, respectively. In cases where the total does not add up to 1761, it indicates that some respondents either answered ’Don’t know’ or refused to provide a response for that variable. The percentages are calculated from the biological mothers who answered the question, excluding those who responded with ’Don’t know’ or ’refused to answer’. Approximately 10.3% of biological mothers resided in the Northern region, with around 33% each in the North East and Upper West regions, and 23.4% in the Upper Eastern region. Roughly two-thirds of mothers identified as Muslim, 38.6% had completed education up to middle school or above, and 77.3% reported being employed. Approximately 93.5% of mothers reported completing at least four ANC visits during their last pregnancy, with a similar percentage delivering at a health facility during their last delivery.

**Table 1:** Characteristics of biological mothers.

Variables	Weighted	Unweighted
	Percent (%)	Total number of responses (N)
**Region**		
Northern	10.3	213
North East	32.9	635
Upper East	23.4	336
Upper West	33.4	577
**Type of residence**		
Rural	42.3	878
Urban	57.7	883
**Religion of caregiver**		
Non-Muslim	33.2	524
Muslim	66.8	1236
**Education status**		
No education or at most primary	61.4	1136
Middle school and above	38.6	625
**Employment status**		
Unemployed	22.7	389
Employed	77.3	1372
**Gender norms**		
Most desirable	28.3	461
Desirable	24.4	442
Not desirable	24.4	423
Not at all desirable	22.9	435
**Number of ANC visits**		
Less than 4 ANC visits	6.5	112
4 or more ANC visits	93.5	1589
**Place of delivery**		
At home	6.5	131
At a hospital/govt health facility/ maternity home	93.5	1629

Note: In instances where the total number of responses (N) does not equal 1761, it indicates that some respondents either answered ’Don’t know’ or refused to provide a response for that variable. The percentages are calculated from the biological mothers who answered the question, excluding those who responded with ’Don’t know’ or ’refused to answer’.

**Table 2:** Months caregiver waited before introducing anything other than breastmilk, such as formula, plain water, cereals, or other mashed foods to the youngest child.

Variables	Weighted	Unweighted
	Percent (%)	Total number of responses (N)
**Exclusively breastfed**		
Less than 6 months	19.2	319
For 6 months	75	1301
Beyond 6 months	5.9	106

Note: In instances where the total number of responses (N) does not equal 1761, it indicates that some respondents either answered ’Don’t know’ or refused to provide a response for that variable. The percentages are calculated from the biological mothers who answered the question, excluding those who responded with ’Don’t know’ or ’refused to answer’.

All biological mothers reported breastfeeding their children. Around 75% of biological mothers indicated waiting for six months before introducing complementary food other than breast milk. Meanwhile, 19.2% of biological mothers waited for less than six months, and 5.9% waited beyond six months before introducing complementary food to their children.

In general, 74.9% of biological mothers stated that the majority of families in their communities introduce babies to foods other than breast milk at six months of age (Table 3). Approximately 95.9% expressed their willingness to encourage friends or family to wait six months before introducing any liquids or other foods to their babies. Around 88.1% reported that if they were to have a baby in the future, they would feel confident in their ability to exclusively breastfeed their baby for six months if they chose to do so.

**Table 3:** Behavioral determinants of EBF.

Variables	Weighted	Unweighted
	Percent (%)	Total number of responses (N)
**Social norms: Age at which families in the communities introduce complementary foods**		
Before or beyond 6 months	25.1	383
At 6 months	74.9	1181
**Positive attitude (approval): Would recommend friends/family wait six months before introducing complementary foods**		
No	4.1	74
Yes	95.9	1656
**Caregiver’s self-efficacy: Confidence in ability to introduce complementary food at 6 months**		
Not at all confident/Somewhat confident	11.9	206
Very Confident	88.1	1546

Note: In instances where the total number of responses (N) does not equal 1761, it indicates that some respondents either answered ’Don’t know’ or refused to provide a response for that variable. The percentages are calculated from the biological mothers who answered the question, excluding those who responded with ’Don’t know’ or ’refused to answer’.

### Factors associated with exclusively breastfeeding for the first six months

The regression models presented in Tables 4 and 5 utilize a sample of 1,448 biological mothers. Participants with missing data on either the dependent variable or any independent variables included in Table 4, Adjusted Model 3, were excluded from the analysis. The Unadjusted Model 1 presented in Table 4, displays a univariate analysis of factors associated with EBF practice for the first six months among biological mothers. Notably, all factors, except for the location of the caregiver’s residence (rural/urban), exhibited a significant (P < 0.1) effect on EBF practice. The percentage of biological mothers who reported practicing EBF for the first six months was more likely to be reported in the Upper West region. Muslim biological mothers were less likely to exclusively breastfeed their child for the first six months compared to non-Muslims. Older, educated, and employed biological mothers showed a higher likelihood of practicing EBF. EBF rates were higher among biological mothers who reported attending four or more ANC visits during their pregnancy as well as those who delivered at a health facility. Additionally, behavioral determinants of EBF, such as social norms around EBF, self-efficacy to practice the behavior, and approval of the behavior, were significantly associated with EBF practice.

**Table 4:** Factors associated with EBF for six months, logistic regression.

	[Unadjusted Model 1]	[Adjusted Model 1] Demographic covariates	[Adjusted Model 2] Demographic+ Care seeking covariates	[Adjusted Model 3] Demographic + Care seeking + Behavioral covariates
Wald test: p-values			0.07	<0.001
**VARIABLES**	OR	aOR	aOR	aOR
(ref = North East) Region = Northern region	0.981	1.055	1.062	1.361
	(0.588–1.638)	(0.666–1.671)	(0.691–1.632)	(0.927–1.997)
Region = Upper East	0.925	0.666*	0.625**	1.254
	(0.576–1.486)	(0.420–1.055)	(0.395–0.989)	(0.803–1.960)
Region = Upper West	1.651*	1.516	1.434	1.404
	(0.928–2.938)	(0.901–2.551)	(0.875–2.349)	(0.832–2.369)
(ref = Rural) Location = Urban	0.973			
	(0.679–1.396)			
Age of caregiver	1.021*	1.024**	1.024**	1.032**
	(0.998–1.043)	(1.001–1.047)	(1.001–1.047)	(1.006–1.059)
(ref = Non-Muslim) Religion = Muslim	0.635*	0.615**	0.604**	0.657**
	(0.391–1.032)	(0.410–0.922)	(0.401–0.908)	(0.434–0.994)
(ref = No education or at most primary) Education = Middle school or above	1.381**	1.324*	1.299*	1.330*
	(1.020–1.868)	(0.974–1.799)	(0.954–1.769)	(0.968–1.828)
(ref = Not employed) Employment = Employed	1.369*	1.295	1.296	1.368
	(0.943–1.986)	(0.899–1.867)	(0.901–1.863)	(0.875–2.140)
(ref = Most desirable) Caregiver- Gender equitable index quartiles, Desirable	1.377	1.539**	1.538**	1.453
	(0.919–2.063)	(1.001–2.365)	(1.009–2.343)	(0.862–2.451)
Caregiver- Gender equitable index quartiles, Not desirable	1.540**	1.871***	1.871***	1.074
	(1.069–2.220)	(1.302–2.688)	(1.291–2.711)	(0.674–1.710)
Caregiver- Gender equitable index quartiles, Not at all desirable	1.086	1.391	1.408	0.840
	(0.718–1.643)	(0.919–2.107)	(0.931–2.130)	(0.518–1.362)
Wealth score- standardized	1.117*	1.101	1.094	1.030
	(0.979–1.275)	(0.935–1.296)	(0.932–1.285)	(0.875–1.214)
(ref = <4 ANC visits) ANC visits = 4 or above	1.481*		1.369	1.279
	(0.942–2.328)		(0.851–2.201)	(0.754–2.171)
(ref = At home) Place of delivery = Hospital	1.656**		1.512*	1.159
	(1.031–2.660)		(0.940–2.431)	(0.682–1.970)
(ref = Less than or more than 6 months) Age at which communities introduce complementary food 6 months	8.201***			6.336***
	(5.756–11.68)			(4.412–9.098)
(ref = No) Confidence to introduce complementary food at 6 months = Yes	11.23***			6.837***
	(7.076–17.84)			(4.126–11.33)
(ref = No) Encourage friends/family to wait six months before introducing complementary food = Yes	46.60***			10.49***
	(15.65–138.7)			(4.131–26.62)
Observations	1,448	1,448	1,448	1,448

Note: a. * indicates p value < or = .10

** indicates p value < or = .05

*** indicates p value < or = .01.

b. OR indicates Odds Ratio, aOR indicates Adjusted Odds Ratio

c. Please refer to S1 Table, where we present ORs for unadjusted Model 1 and adjusted Models 1 and 2 without restricting the sample to that of Adjusted Model 3. The change in the sample size does not alter the direction of the odds ratios and the overall interpretation of results.

**Table 5:** Factors associated with suboptimal breastfeeding (EBF for less than or beyond six months), multinomial logistic regression.

	Adjusted Model Demographic + Care seeking + Behavioral covariates	
VARIABLES	RRR	RRR
	(ref = EBF for six months) Outcome: EBF for less than six months	(ref = EBF for six months) Outcome: EBF beyond six months.
(ref = North East) Region = Northern region	0.758	0.767
	(0.472–1.216)	(0.414–1.424)
Region = Upper East	1.342	0.368**
	(0.803–2.242)	(0.140–0.966)
Region = Upper West	0.944	0.470
	(0.511–1.745)	(0.189–1.171)
(ref = Rural) Location = Urban	1.563**	0.957
	(1.008–2.421)	(0.579–1.583)
Age of caregiver	0.957***	0.988
	(0.930–0.986)	(0.945–1.033)
(ref = non-Muslim) Religion = Muslim	1.749**	1.241
	(1.065–2.873)	(0.755–2.039)
(ref = No education or at most primary) Education = Middle school or above	0.721*	0.778
	(0.492–1.056)	(0.421–1.436)
(ref = Not employed) Employment = Employed	0.679*	0.912
	(0.433–1.066)	(0.412–2.023)
(ref = Most desirable) Caregiver- Gender equitable index quartiles, Desirable	0.840	0.689
	(0.434–1.624)	(0.363–1.307)
Caregiver- Gender equitable index quartiles, Not desirable	1.075	0.793
	(0.617–1.873)	(0.320–1.967)
Caregiver- Gender equitable index quartiles, Not at all desirable	1.298	1.209
	(0.752–2.241)	(0.517–2.825)
Wealth factor score- standardized	0.899	1.006
	(0.777–1.041)	(0.729–1.387)
(ref = <4 ANC visits) ANC visits = 4 or above	0.678	1.126
	(0.377–1.218)	(0.472–2.685)
(ref = At home) Place of delivery = Hospital	0.615	0.909
	(0.336–1.125)	(0.385–2.144)
(ref = Less than or more than 6 months) Age at which communities introduce complementary food = 6 months	0.154***	0.140***
	(0.103–0.232)	(0.0910–0.214)
(ref = No) Confidence to introduce complementary food at 6 months = Yes	0.0810***	0.423**
	(0.0504–0.130)	(0.190–0.942)
Observations	1,448	1,448

* Note: a. * indicates p value < or = .10

** indicates p value < or = .05

*** indicates p value < or = .01.

b. RRR indicates Relative Risk Ratios

c. Please refer to S2 Table, where we present RRRs for Table 5 without restricting the sample to that of Table 4, Adjusted Model 3. The change in the sample size does not alter the direction of the odds ratios and the overall interpretation of results.

The multivariate logistic regression model (Adjusted Model 3) in Table 4 identified independent factors associated with EBF practice among biological mothers. The odds of practicing EBF for the first six months increased (AOR:1.03, 95% CI,1.01–1.06) with the mother’s age. Muslim biological mothers had 44% lower odds (AOR: 0.66, 95% CI, 0.43–0.99) of practicing EBF compared to non-Muslim biological mothers. Educated biological mothers with middle school or higher education were 1.3 times as likely to practice EBF (AOR:1.3, 95% CI, 0.97–1.83) as those with no education or at most primary education, although these effects are marginal given the confidence interval. Biological mothers who reported that families in their communities practiced EBF for the first six months were 6 times (AOR: 6.3, 95% CI, 4.41–9.10) as likely to practice EBF as biological mother who reported families in their community practicing EBF for less than or more than six months. Similarly, biological mothers who were confident in their ability to exclusively breastfeed if they were to have a child in the future were approximately 6.8 times (AOR: 6.8, 95% CI, 4.13–11.33) as likely to report practicing EBF as those who were not confident. Moreover, biological mothers who approved of EBF practice by stating that they would recommend EBF for the first six months to their friends and family were around 10 times (AOR: 10.5, 95% CI, 4.13–26.6) as likely to report practicing EBF as those stating they would not encourage.

### Factors associated with suboptimal EBF practices

The multivariate multinomial logistic regression model (Table 5) identified factors correlated with EBF for less than six months (introducing complementary food before six months) and EBF beyond six months (not introducing complementary foods at six months).

There was minimal overlap in the factors linked to EBF for less than six months and EBF beyond six months. Older age, higher education, employment, and delivery at a health facility reduced the likelihood of EBF practice for less than six months compared to EBF for the first six months. On the other hand, affiliation with the Muslim religion and residing in urban area were associated with a higher likelihood of EBF for less than six months compared to our reference category. Additionally, supportive social norms on EBF for the first six months and possessing confidence in one’s ability to EBF for six months were statistically associated with the reduced likelihood of practicing EBF for less than six months compared to EBF for six months. None of these factors except the two behavioral determinants (social norms and self-efficacy) were statistically significant and indicated a reduced likelihood of practicing EBF beyond six months as compared to EBF for six months.

## Discussion

The present research investigated the extent to which biological mothers of children under five in Northern Ghana ASBC project geography adhere to the recommended EBF duration for the first six months. It also explored the factors linked to adherence to this recommended duration. The study identified perceived social norms, confidence in the ability to follow the recommended behavior, and approval of the behavior as significant factors influencing biological mothers’ practice of EBF for the first six months. Additionally, the religion and education level of biological mothers were identified as socio-demographic factors associated with adherence to the recommended EBF duration.

One key takeaway from the study is the significant influence of social norms on biological mothers’ EBF practices. The observed association between adherence to the recommended EBF duration and social norms is supported by other studies [26, 27] and highlights the importance of community and cultural contexts in shaping biological mothers’ behaviors. Based on existing evidence around behavior change techniques, mechanisms of action that can support improved norms include social comparison and information about others’ approval of behavior [28]. For example, this might involve disseminating success stories of mothers who have effectively practiced EBF for the recommended duration. In the context of Northern Ghana, mothers-in-law were found to be important gatekeepers to ensure support for breastfeeding in general, inclusive of EBF [11, 20, 29]. This finding may also suggest that social support networks and peer/familial group-based interventions could be important, given the influence of social norms on EBF behaviors.

Furthermore, the study identified confidence in the ability to follow recommended EBF behavior as an important factor, also found in other studies [27, 30]. Interventions to target improved self-efficacy and agency include disseminating information about how to perform the behavior and the benefits of EBF, demonstrations of the behavior, empowering biological mothers with the problem-solving skills needed to adopt and sustain these practices within their contexts, and addressing social norms at the community level that underpin women’s agency.

Recognition of the link between biological mothers’ approval of EBF in predicting EBF practices is vital to programs that wish to increase the uptake of this behavior. Programs need to emphasize the advantages of EBF and supporting breastfeeding mothers. This could be directly through improved provider counseling at health facilities, especially ANC touchpoints, as it offers a gateway to other important maternal and child health information and services, as well as through establishing or leveraging existing peer support networks (e.g., mother-to-mother support groups, village savings, and loan associations, etc.) as a platform to share crucial information and skills related to EBF and infant and young child feeding in general. Additionally, fostering open communication between healthcare professionals and biological mothers is essential to address concerns, offer ongoing support, and enhance their approval of recommended behaviors.

We note that the study also highlights the predictive role of religion, and to a lesser degree education, in biological mothers’ adherence to the recommended EBF duration. Media campaigns may need to be tailored to the religious and educational backgrounds of biological mothers to be effective in promoting and sustaining optimal EBF durations and breastfeeding practices. Additionally, fully understanding the characteristics of Muslim families that may influence EBF behaviors should be further explored.

There are limitations to the study. Firstly, this was a baseline survey for a social and behavioral change project across multiple health areas. One limitation, given this, is that some of the more EBF specific determinants were not included due to time limitations. These include questions on maternal biological factors such as mother’s body mass index at the time of birth, mode of delivery, birth complications, experiences at the birth facility (rooming, skin-to-skin care, infant feeding difficulties), type of complementary foods introduced to the infants, mother’s employment and/or workplace policies regarding maternity leave and breastfeeding, and mother’s exposure to formula marketing directly at stores or in the digital space, or via healthcare workers [31, 32]. We recognize the effect of structural barriers on individual and social-level determinants of EBF [6, 10]. However, we were not able to directly account for structural barriers to EBF outside of the demographic controls. We anticipate that structural barriers such as national-level employment and maternity care policies are highly correlated with demographic factors like education and wealth, both of which were included as covariates in the model. Also, only 5.85% of study participants reported having a full-time salaried job, where maternity care policies are more prevalent. Other study participants coded as being employed were either employed part time receiving in-kind or cash payments, self-employed, students with full- or part-time jobs or served as an apprentice. Second, the questions were framed as recalling information about the last pregnancy, delivery, or last childbirth within the past five years, and the extended recall period may have affected the precision of respondents’ answers. To enhance accuracy, we limited the sample to only female biological mothers, considering their proximity to the child. Additionally, questions regarding EBF practices, care-seeking behavior, and behavioral determinants of EBF were referenced either to the last delivery, last pregnancy, or last childbirth. Last delivery and childbirth can be two separate events for some biological mothers which may lead biological mothers to refer to different events while responding to these questions. Third, it Is important to note that 80.9% respondents reported practicing EBF for six months or beyond, which appears to be an overestimation compared to the Ghana Demographic and Health Survey (DHS) (2022) results indicating that 53% of children aged 0–5 months were exclusively breastfeed (only breast milk) the previous day of the survey, nationally. However, the percentage of children aged 0–5 months exclusively breastfed in North Ghana was above the national average except for the Upper East region; Northern region (73.2%), North East (65.5%), Upper West (68.1%), and Upper East (51.8%). The overestimation may be attributed to social desirability bias in our data. The differences may also arise from the longer recall period in our study (up to five years) compared to the previous day’s breastfeeding status recall in the DHS. Both retrospective studies with longer recall duration and point in time data on EBF have its own merits and demerits, Greiner (2014) suggests the most effective method for acquiring lifelong data on EBF could involve interviewing all mothers with infants under seven (or possibly nine) months old. During these interviews, mothers would be asked to provide details about each locally common early supplement given to their baby and when it was initially introduced [33]. Another limitation pertains to the external validity of the findings. The sampling weight is calculated as the inverse of the probability of choosing households in each EA. Therefore, our findings are representative of EAs selected in the sample and not nationally representative. Finally, we also note that as with many social and behavioral surveys, social desirability bias may influence the responses of participants.

Despite these limitations, the study’s primary strength lies in its incorporation of behavioral determinants of optimal EBF duration within the model and our extension to examining predictors of mothers who EBF for less than or longer than six months. To our knowledge, very few research studies specific to Ghana have delved into the influence of social norms and self-efficacy on the optimal duration of EBF. An important contribution is made by differentiating between EBF for less than six months and beyond six months, allowing for the identification of factors that predict suboptimal exclusive breastfeeding behaviors within these distinct timeframes and regions.

## Conclusions

In conclusion, this research assessed factors influencing EBF duration among biological mothers of children under five in four regions of Northern Ghana. The research provided insights into adherence to the recommended six-month EBF duration and the associated factors. Social norms, confidence in adopting recommended behaviors, and approval of such behaviors emerged as pivotal factors shaping biological mothers’ EBF practices for the recommended duration. Furthermore, the study identified the caregiver’s religion, and education level as significant socio-demographic predictors influencing adherence to the recommended EBF duration. While some of the more EBF-specific determinants were not collected as a part of the study, the findings still underscore the multifaceted nature of influences on EBF practices, providing inputs for targeted interventions aimed at promoting optimal breastfeeding behaviors among biological mothers in Northern Ghana. Improved uptake of EBF has the potential to improve child development via adequate nutritional intake, thereby reducing child mortality and morbidity.

## Supporting information

S1 Table Factors associated with EBF for six months, logistic regression. (DOCX)

S2 Table Factors associated with suboptimal breastfeeding (EBF for less than or beyond six months), multinomial logistic regression. (DOCX)

S1 Checklist Inclusivity in global research. (DOCX)

## References

[1] Anin SK, Saaka M, Fischer F, Kraemer A. Association between infant and young child feeding (IYCF) indicators and the nutritional status of children (6–23 months) in northern Ghana. Nutrients. 2020;12(9):2565. doi: 10.3390/nu12092565 32847027 PMC7551146

[2] Kramer MS, Kakuma R. Optimal duration of exclusive breastfeeding. Cochrane database of systematic reviews. 2012(8). doi: 10.1002/14651858.CD003517.pub2 22895934 PMC7154583

[3] Victora CG, Bahl R, Barros AJ, França GV, Horton S, Krasevec J, et al. Breastfeeding in the 21st century: epidemiology, mechanisms, and lifelong effect. The lancet. 2016;387(10017):475–90. doi: 10.1016/S0140-6736(15)01024-7 26869575

[4] Ghana Statistical Service (GSS) and ICF. 2023. Ghana Demographic and Health Survey 2022: Key Indicators Report. Accra, Ghana, and Rockville, Maryland, USA: GSS and ICF.

[5] Dujin B. Caregivers Adherence to the WHO Breastfeeding Guideline and Nutritional Status of Children under Five Years in Northern Region of Ghana.. Food Science and Quality Management, 86 2019

[6] Pérez-Escamilla R, Tomori C, Hernández-Cordero S, Baker P, Barros AJD, Bégin F, et al. Breastfeeding: crucially important, but increasingly challenged in a market-driven world. Lancet. 2023;401(10375):472–85. doi: 10.1016/S0140-6736(22)01932-8 36764313

[7] Asimaki E, Dagla M, Sarantaki A, Iliadou M. Main Biopsychosocial Factors Influencing Breastfeeding: a Systematic Review. Maedica (Bucur). 2022;17(4):955–62. doi: 10.26574/maedica.2022.17.4.955 36818247 PMC9923068

[8] Pérez-Escamilla R, Martinez JL, Segura-Pérez S. Impact of the Baby-friendly Hospital Initiative on breastfeeding and child health outcomes: a systematic review. Matern Child Nutr. 2016;12(3):402–17. doi: 10.1111/mcn.12294 26924775 PMC6860129

[9] Patil DS, Pundir P, Dhyani VS, Krishnan JB, Parsekar SS, D’Souza SM, et al. A mixed-methods systematic review on barriers to exclusive breastfeeding. Nutrition and Health. 2020;26(4):323–46. doi: 10.1177/0260106020942967 33000699

[10] Likhite N, Adossi D, Oot L, Dain ASL, Tharaney M, Nanama S. Factors Influencing the Practice of Exclusive Breastfeeding in West and Central Africa: Curr Dev Nutr. 2022 Jun 14;6(Suppl 1):850. doi: 10.1093/cdn/nzac065.034 eCollection 2022 Jun.

[11] Ziblim S, Yidana A., & Seidu I. Family Related Factors Influencing Exclusive Breastfeeding in Rural Northern Ghana: A Qualitative Analysis.. NUMID Horizon: An International Journal of Nursing and Midwifery, 3(1). 2020.

[12] Dun-Dery EJ, Laar AK. Exclusive breastfeeding among city-dwelling professional working mothers in Ghana. International breastfeeding journal. 2016;11(1):1–9. doi: 10.1186/s13006-016-0083-8 27602050 PMC5012076

[13] Mogre V, Dery M, Gaa PK. Knowledge, attitudes and determinants of exclusive breastfeeding practice among Ghanaian rural lactating mothers. International breastfeeding journal. 2016;11(1):1–8. doi: 10.1186/s13006-016-0071-z 27190546 PMC4869336

[14] Agbozo F, Ocansey D, Atitto P, Jahn A. Compliance of a Baby-Friendly Designated Hospital in Ghana With the WHO/UNICEF Baby and Mother-Friendly Care Practices. J Hum Lact. 2020;36(1):175–86. doi: 10.1177/0890334419848728 31112053

[15] Al-Thubaity DD, Alshahrani MA, Elgzar WT, Ibrahim HA. Determinants of High Breastfeeding Self-Efficacy among Nursing Mothers in Najran, Saudi Arabia. Nutrients. 2023;15(8). doi: 10.3390/nu15081919 37111138 PMC10145845

[16] Sultana M, Dhar S, Hasan T, Shill LC, Purba NH, Chowdhury AI, et al. Knowledge, attitudes, and predictors of exclusive breastfeeding practice among lactating mothers in Noakhali, Bangladesh. Heliyon. 2022;8(10). doi: 10.1016/j.heliyon.2022.e11069 36276726 PMC9578980

[17] Mohammed S, Yakubu I, Fuseini A-G, Abdulai A-M, Yakubu YH. Systematic review and meta-analysis of the prevalence and determinants of exclusive breastfeeding in the first six months of life in Ghana. BMC Public Health. 2023;23(1):920. doi: 10.1186/s12889-023-15758-w 37208682 PMC10199593

[18] Nukpezah RN, Nuvor SV, Ninnoni J. Knowledge and practice of exclusive breastfeeding among mothers in the tamale metropolis of Ghana. Reprod Health. 2018;15(1):140. doi: 10.1186/s12978-018-0579-3 30134962 PMC6106742

[19] Aryeetey RN, Tay M. Compliance Audit of Processed Complementary Foods in Urban Ghana. Front Public Health. 2015;3:243. doi: 10.3389/fpubh.2015.00243 26579505 PMC4621382

[20] Tampah-Naah AM, Kumi-Kyereme A, Amo-Adjei J. Maternal challenges of exclusive breastfeeding and complementary feeding in Ghana. PLoS One. 2019;14(5):e0215285. doi: 10.1371/journal.pone.0215285 31048865 PMC6497241

[21] WHO. Complementary feeding. Available from: https://www.who.int/health-topics/complementary-feeding#tab=tab_1.

[22] WHO. WHO Guideline for complementary feeding of infants and young children 6–23 months of age.. Geneva: World Health Organization; 2023. Licence: CC BY-NC-SA 3.0 IGO.; 2023. 37871145

[23] Edgar Cooke SH, Andy McKay The Ghana Poverty and Inequality Report: Using the 6th Ghana Living Standards Survey. 2016.

[24] USAID. Country Development Cooperation Strategy (CDCS). Available from: https://www.usaid.gov/sites/default/files/2022-05/CDCS-Ghana-August-2025x.pdf.

[25] Zissette S, Tolley EE, Martinez A, Roberts ST, Palanee-Phillips T, Montgomery ET. Measuring Effects of Counseling to Increase Pre-Exposure Prophylaxis Adherence and Partner Support in South Africa Using the Healthy Relationship Assessment Tool. Global Health: Science and Practice. 2023;11(5). doi: 10.9745/GHSP-D-22-00075 37903586 PMC10615234

[26] Bicchieri C, Das U, Gant S, Sander R. Examining norms and social expectations surrounding exclusive breastfeeding: Evidence from Mali. World Development. 2022;153:105824.

[27] Zhang Z, Zhu Y, Zhang L, Wan H. What factors influence exclusive breastfeeding based on the theory of planned behaviour. Midwifery. 2018;62:177–82. doi: 10.1016/j.midw.2018.04.006 29684797

[28] Arigo D, Mogle JA, Brown MM, Pasko K, Travers L, Sweeder L, et al. Methods to assess social comparison processes within persons in daily life: a scoping review. Frontiers in Psychology. 2020;10:2909. doi: 10.3389/fpsyg.2019.02909 32038352 PMC6987244

[29] Adda L, Opoku-Mensah K, Dako-Gyeke P. “Once the child is delivered, he is no more your baby,” Exclusive Breastfeeding experiences of first-time mothers in Kassena-Nankana Municipality, Ghana-a qualitative study. BMC Pregnancy and Childbirth. 2020;20(1):1–9. 10.1186/s12884-020-03272-5 PMC7526357 32993563

[30] Nguyen NT, Prasopkittikun T, Payakkaraung S, Vongsirimas N. Factors predicting six-month exclusive breastfeeding among mothers in Ho Chi Minh City, Vietnam. Journal of Health Research. 2021;36(2):219–30.

[31] Segura-Pérez S, Richter L, Rhodes EC, Hromi-Fiedler A, Vilar-Compte M, Adnew M, et al. Risk factors for self-reported insufficient milk during the first 6 months of life: A systematic review. Matern Child Nutr. 2022;18 Suppl 3(Suppl 3):e13353. 10.1111/mcn.13353 PMC9113468 35343065

[32] Pérez-Escamilla R, Hromi-Fiedler A, Rhodes EC, Neves PAR, Vaz J, Vilar-Compte M, et al. Impact of prelacteal feeds and neonatal introduction of breast milk substitutes on breastfeeding outcomes: A systematic review and meta-analysis. Matern Child Nutr. 2022;18 Suppl 3(Suppl 3):e13368. doi: 10.1111/mcn.13368 35489107 PMC9113480

[33] Greiner T. Exclusive breastfeeding: measurement and indicators. International Breastfeeding Journal. 2014;9(1):18. doi: 10.1186/1746-4358-9-18 25349624 PMC4209171

