# Supplementary material for: Factors associated with exclusive breastfeeding for the first six months among caregivers of children under five years in northern Ghana: A cross-sectional study
Source: PLOS Glob Public Health. 2024 Nov 14;4(11):e0003887. doi: 10.1371/journal.pgph.0003887 (PMC11563355; doi:10.1371/journal.pgph.0003887)
Supplement: S1 Table — (DOCX) [file pgph.0003887.s001.docx]

*S1 Table. Factors associated with EBF for six months, logistic regression*

|  | [Unadjusted Model 1] | [Adjusted Model 1]  Demographic covariates | [Adjusted Model 2]  Demographic+  Care seeking  covariates | [Adjusted Model 3]  Demographic + Care seeking  + Behavioral covariates |
| --- | --- | --- | --- | --- |
| Wald test: p-values |  |  | 0.02 | <0.001 |
| **VARIABLES** | OR | aOR | aOR | aOR |
| (ref= North East) Region= Northern region | 0.966 | 1.027 | 1.060 | 1.361 |
|  | (0.570 - 1.636) | (0.643 - 1.642) | (0.674 - 1.665) | (0.927 - 1.997) |
| Region= Upper East | 0.985 | 0.739 | 0.720 | 1.254 |
|  | (0.597 - 1.627) | (0.458 - 1.190) | (0.440 - 1.179) | (0.803 - 1.960) |
| Region= Upper West | 1.700* | 1.515 | 1.447 | 1.404 |
|  | (0.986 - 2.932) | (0.915 - 2.506) | (0.886 - 2.364) | (0.832 - 2.369) |
| (ref=Rural) Location=Urban | 0.956 |  |  |  |
|  | (0.679 - 1.346) |  |  |  |
| Age of caregiver | 1.020** | 1.022** | 1.024** | 1.032** |
|  | (1.002 - 1.037) | (1.003 - 1.042) | (1.004 - 1.044) | (1.006 - 1.059) |
| (ref=Non-Muslim) Religion= Muslim | 0.621** | 0.650** | 0.655** | 0.657** |
|  | (0.392 - 0.983) | (0.443 - 0.954) | (0.444 - 0.967) | (0.434 - 0.994) |
| (ref=No education or at most primary) Education= Middle school or above | 1.322** | 1.257 | 1.234 | 1.330* |
|  | (1.023 - 1.709) | (0.946 - 1.672) | (0.929 - 1.640) | (0.968 - 1.828) |
| (ref=Not employed) Employment= Employed | 1.400** | 1.251 | 1.273 | 1.368 |
|  | (1.019 - 1.922) | (0.905 - 1.730) | (0.916 - 1.768) | (0.875 - 2.140) |
| (ref= Most desirable) Caregiver- Gender equitable index quartiles, Desirable | 1.300 | 1.486* | 1.438* | 1.453 |
|  | (0.888 - 1.901) | (0.998 - 2.212) | (0.972 - 2.126) | (0.862 - 2.451) |
| Caregiver- Gender equitable index quartiles, Not desirable | 1.352* | 1.720*** | 1.696*** | 1.074 |
|  | (0.953 - 1.917) | (1.213 - 2.440) | (1.178 - 2.441) | (0.674 - 1.710) |
| Caregiver- Gender equitable index quartiles, Not at all desirable | 0.998 | 1.305 | 1.262 | 0.840 |
|  | (0.678 - 1.467) | (0.888 - 1.919) | (0.855 - 1.862) | (0.518 - 1.362) |
| Wealth score- standardized | 1.117* | 1.097 | 1.086 | 1.030 |
|  | (0.997 - 1.252) | (0.940 - 1.281) | (0.930 - 1.268) | (0.875 - 1.214) |
| (ref=<4 ANC visits) ANC visits= 4 or above | 1.594** |  | 1.564* | 1.279 |
|  | (1.019 - 2.493) |  | (0.986 - 2.481) | (0.754 - 2.171) |
| (ref=At home) Place of delivery= Hospital | 1.688** |  | 1.422 | 1.159 |
|  | (1.069 - 2.667) |  | (0.872 - 2.320) | (0.682 - 1.970) |
| (ref= Less than or more than 6 months) Age at which communities introduce complementary food 6 months | 8.489*** |  |  | 6.336*** |
|  | (6.100 - 11.81) |  |  | (4.412 - 9.098) |
| (ref=No) Confidence to introduce complementary food at 6 months= Yes | 14.35*** |  |  | 6.837*** |
|  | (9.222 - 22.33) |  |  | (4.126 - 11.33) |
| (ref=No) Encourage friends/family to wait six months before introducing complementary food=Yes | 63.89*** |  |  | 10.49*** |
|  | (21.15 - 193.0) |  |  | (4.131 - 26.62) |
|  |  |  |  |  |
| Observations |  | 1,689 | 1,636 | 1,448 |

Note: a. * indicates p value < or = .10, ** indicates p value < or = .05, *** indicates p value < or = .01.

b. OR indicates Odds Ratio, aOR indicates Adjusted Odds Ratio

c. The number of observations varies for each independent variable listed under Unadjusted Model 1 because these are separate univariate logistic regressions, and the missing data differs across each of these the independent variables.
