# Supplementary material for: Factors associated with exclusive breastfeeding for the first six months among caregivers of children under five years in northern Ghana: A cross-sectional study
Source: PLOS Glob Public Health. 2024 Nov 14;4(11):e0003887. doi: 10.1371/journal.pgph.0003887 (PMC11563355; doi:10.1371/journal.pgph.0003887)
Supplement: S2 Table — (DOCX) [file pgph.0003887.s002.docx]

S2 Table. Factors associated with suboptimal breastfeeding (EBF for less than or beyond six months), multinomial logistic regression

|  | **Adjusted Model**  Demographic + Care seeking  + Behavioral covariates | |
| --- | --- | --- |
| **VARIABLES** | **RRR** | **RRR** |
|  | (ref= EBF for six months)  Outcome: EBF for less than six months | (ref= EBF for six months)  Outcome: EBF beyond six months. |
| (ref= North East) Region= Northern region | 0.743 | 0.778 |
|  | (0.465 - 1.188) | (0.419 - 1.443) |
| Region= Upper East | 1.286 | 0.374** |
|  | (0.775 - 2.133) | (0.143 - 0.981) |
| Region= Upper West | 0.923 | 0.471 |
|  | (0.506 - 1.684) | (0.189 - 1.176) |
| (ref=Rural) Location=Urban | 1.548** | 0.960 |
|  | (1.005 - 2.385) | (0.580 - 1.590) |
| Age of caregiver | 0.957*** | 0.988 |
|  | (0.930 - 0.986) | (0.945 - 1.033) |
| (ref=non-Muslim) Religion= Muslim | 1.750** | 1.244 |
|  | (1.066 - 2.870) | (0.757 - 2.044) |
| (ref=No education or at most primary) Education= Middle school or above | 0.729* | 0.785 |
|  | (0.500 - 1.062) | (0.426 - 1.446) |
| (ref=Not employed) Employment= Employed | 0.670* | 0.915 |
|  | (0.430 - 1.043) | (0.413 - 2.028) |
| (ref= Most desirable) Caregiver- Gender equitable index quartiles, Desirable | 0.799 | 0.691 |
|  | (0.415 - 1.538) | (0.363 - 1.315) |
| Caregiver- Gender equitable index quartiles, Not desirable | 1.060 | 0.804 |
|  | (0.609 - 1.847) | (0.324 - 1.996) |
| Caregiver- Gender equitable index quartiles, Not at all desirable | 1.245 | 1.222 |
|  | (0.730 - 2.125) | (0.522 - 2.859) |
| Wealth factor score- standardized | 0.899 | 1.006 |
|  | (0.777 - 1.039) | (0.729 - 1.388) |
| (ref=<4 ANC visits) ANC visits= 4 or above | 0.699 | 1.118 |
|  | (0.387 - 1.263) | (0.468 - 2.671) |
| (ref=At home) Place of delivery= Hospital | 0.589* | 0.892 |
|  | (0.325 - 1.066) | (0.379 - 2.097) |
| (ref= Less than or more than 6 months) Age at which communities introduce complementary food= 6 months | 0.152*** | 0.140*** |
|  | (0.102 - 0.225) | (0.0911 - 0.216) |
| (ref=No) Confidence to introduce complementary food at 6 months= Yes | 0.0740*** | 0.437** |
|  | (0.0465 - 0.118) | (0.196 - 0.970) |
| Observations | 1,466 | 1,466 |

*** Note: a. * indicates p value < or = .10, ** indicates p value < or = .05, *** indicates p value < or = .01.

b. RRR indicates Relative Risk Ratios
