# Supplementary material for: Factors associated with exclusive breastfeeding for the first six months among caregivers of children under five years in northern Ghana: A cross-sectional study
Source: PLOS Glob Public Health. 2024 Nov 14;4(11):e0003887. doi: 10.1371/journal.pgph.0003887 (PMC11563355; doi:10.1371/journal.pgph.0003887)
Supplement: S1 Checklist — (DOCX) [file pgph.0003887.s003.docx]

Inclusivity in global research

PLOS’ policy on inclusivity in global research aims to improve transparency in the reporting of research performed outside of researchers’ own country or community and ensures that PLOS publications reporting global research adhere to high standards for research ethics and authorship. Authors of relevant research articles may be asked to complete the questionnaire below, which outlines ethical, cultural, and scientific considerations specific to inclusivity in global research. This questionnaire may be requested when researchers have travelled to a different country to conduct research, if research uses samples collected in another country, research with Indigenous populations or their lands, or if research is on cultural artefacts. Researchers travelling to another country solely to use laboratory equipment will not normally be required to complete the questionnaire. However, the questionnaire can be requested at the journal’s discretion for any submission – if you have been requested to complete this questionnaire by the PLOS journal you submitted to, please do so.

Please complete the questionnaire below and include this as a Supporting Information file with your manuscript. Note that if your paper is accepted for publication, this checklist will be published with your article in the supporting information files. Please ensure that you reference the checklist in the main body of your manuscript. We suggest adding a subsection ‘Inclusivity in global research’ to your Methods section and adding the following sentence: “Additional information regarding the ethical, cultural, and scientific considerations specific to inclusivity in global research is included in the Supporting Information (SX Checklist)”

The questions have been designed to be applicable to a wide range of study types, and there are subsections for both human subjects research and non-human subjects research. If any of the questions are not relevant to your research please mark them as “N/A” as appropriate.

**Ethical considerations, permits and authorship**

*This section is applicable to all research types.*

Provide details as to who granted permissions and/or consent for the study to take place in the Methods section of your manuscript. This should include the names of **all** ethics boards, governmental organizations, community leaders or other bodies that provided approval for the study. If individuals provided approval refer to these people by their role or title but do not list their name(s).

Reported on page number: Page 6

If there were any deviations from the study protocol after approval was obtained please provide details of these changes in the Methods section of your manuscript.
Did this study involve local collaborators that are residents of the country where the research was conducted or members of the community studied? If you do not have any authors from said communities, please provide an explanation for this below.

Reported on page number: No deviations reported

Yes.

Everyone listed as an author should meet PLOS’ criteria for authorship and all individuals who meet these criteria should be included in the author byline, rather than the acknowledgements. For further information please see the journal’s Authorship Policy.

**Human subjects research (e.g. health research, medical research, cross-cultural psychology)**

Did you obtain written informed consent from a representative of the local community or region before the research took place? How did you establish who speaks for the community? Details of written informed consent obtained from study participants should be reported separately in the Methods section of your manuscript.

The Ghana Health Service / Health Promotion Division engaged District Health Directors of districts covered by the study to inform them about the study and solicit support for the data collection teams. In addition, District Health Promotion Officers and Community Health Volunteer (CHVs) of selected enumeration areas (EAs) supported community entry activities. CHVs were key to successful community entry across all communities by liaising with data collection teams and scheduling meetings with community chiefs or opinion leaders. This strategy ensured that there were no delays in conducting community entry in the selected EAs. Enumeration teams explained the purpose of their visit and sought verbal permission to begin the data collection in their respective areas.

How did members of the local community provide input on the aims of the research investigation, its methodology, and its anticipated outcome(s)?

The Accelerating Social and Behavior Change (ASBC) project uses participatory action media methods to design its intervention. While we did not gather local community inputs in the study design, the project collected the local community inputs of which this study was a part.

When engaging with the local community, how did you ensure that the informed consent documents and other materials could be understood by local stakeholders?

The research team took several approaches to ensure potential participants understood the information provided in the consent form including their role in the research, risks, and benefits. 1) The team used the most appropriate language that the potential participant understood and was able to interact with the data collector. 2) Enumerators checked for understanding by the potential participant by asking if they have any questions and spent as much time needed answering questions. 3) If the participant agreed to participate in the study, the enumerator asked them to sign/thumb print the consent form, confirming that the participant’s questions were answered and that the participant voluntarily agreed to take part in the research. For adolescents/young persons (less than 18 years), parental/guardian permission was requested, followed by child assent. For young persons under 18 years of age who were married, pregnant, or mothers, personal consent on account of being emancipated minors (children who have assumed adult responsibilities) was sought.

For all activities, enumerators emphasized that participation was voluntary, that there would be no penalty if the individual decided not to participate, and that participants could stop taking part or withdraw from the research/interview at any time.

Will the findings of the research be made available in an understandable format to stakeholders in the community where the study was conducted (e.g. via a presentation, summary report, copies of publications, etc.)? Please provide details of how this will be achieved.

The findings were disseminated in the form of a report to USAID. They were also shared with implementing partners who work with the communities in the study areas.

**Non-human subjects research using specimens/ animals collected as part of the study, or those housed in archival collections. Examples include archaeology, paleontology, botany and zoology.**

Did the permission you obtained from a local authority to perform the study include an agreement on access to outputs and benefit sharing? This may include procedures to enable fair distribution of the benefits and resources arising from the research performed. Please include any details of Prior Informed Consent and Benefit Sharing Agreements obtained. These may be required by field-specific regulations, for example the Convention on Biological Diversity (CBD) and the associated Nagoya Protocol.

Not applicable

If the material used in your study was imported, please A) provide the year it was imported and B) indicate whether permits were obtained to import/export the materials used, C) provide details of any permits obtained. If this information is not available, please indicate this.

Not applicable

If you used archival specimens, please state how the material used in your study was acquired by the institute it is held in and provide details of any permits obtained for the original excavations/ sample collection. If this information is not available, please indicate this.

Not applicable

How was the potential cultural significance of the materials collected in your study to local communities considered in your research design? Were Indigenous peoples and/or local researchers and institutions involved with archaeological excavations / collection of specimens? If so, please provide a description of their involvement.

Not applicable

If your manuscript includes photographs of human remains please indicate whether authors obtained permission from descendants or affiliated cultural communities to do so.

Not applicable
